# Quantifying inter-organelle membrane contact sites using proximity ligation assay in fixed optic nerve sections

**DOI:** 10.1016/j.exer.2021.108793

**Published:** 2021-12

**Authors:** Jared Ching, Andrew Osborne, Richard Eva, Julien Prudent, Patrick Yu-Wai-Man

**Affiliations:** a John van Geest Centre for Brain Repair, Department of Clinical Neurosciences, University of Cambridge, Cambridge, UK; b Medical Research Council Mitochondrial Biology Unit, University of Cambridge, Cambridge, UK; c Department of Ophthalmology, Addenbrooke's Hospital, Cambridge, UK; d Ikarovec Ltd, Norwich Innovation Centre, Norwich, UK; e Moorfields Eye Hospital, London, UK; f UCL Institute of Ophthalmology, University College London, London, UK

**Keywords:** Membrane contact sites, Proximity ligation assay, Protrudin, Optic nerve, Retinal ganglion cells

## Abstract

Membrane contact sites (MCS) play crucial roles in cell physiology with dysfunction in several MCS proteins being linked with neurological and optic nerve diseases. Although there have been significant advances in imaging these interactions over the past two decades with advanced electron microscopy techniques, super-resolution imaging and proximity-dependent fluorescent reporters, a technique to observe and quantify MCS in mammalian optic nerve tissues has not yet been reported. We demonstrate for the first time that proximity ligation assay (PLA), a technique already used in mammalian cell lines, can be used as an efficient method of quantifying inter-organelle contact sites, namely mitochondria-endoplasmic reticulum (ER) and mitochondria-late-endosomes, in mammalian optic nerve tissues treated with adeno-associated virus (AAV) gene therapy with wild-type or phosphomimetic (active) protrudin. PLA utilises complementary single-stranded DNA oligomers bound to secondary antibodies that hybridise and complete a circular piece of DNA when the primary antibodies of interest interact. These interactions can be detected by amplifying the circular DNA and adding fluorescent probes. We show that PLA is a useful method that can be used to quantify MCS in optic nerve tissues. We have found that upregulation of protrudin with gene therapy significantly increases the number of mitochondria-ER and mitochondria-Rab7-late endosomes contact sites in optic nerves.

## 1 Introduction

Membrane contact sites (MCS) form between membrane-bound cellular organelles in order to facilitate communication and perform specific functions (Elbaz and Schuldiner, 2011). MCS are known to be crucial in cell physiology where a number of MCS proteins have been linked to neurodegenerative diseases that can also affect the optic nerve, such as spastic paraplegia (protrudin, seipin, and spastin), vesicle-associated membrane protein (VAMP)-associated protein (VAP), and retinal dystrophy with leukodystrophy (Acyl-CoA-binding domain-containing protein 5 (ACBD5)) (Krols et al., 2016). We now have a better understanding of the numerous processes occurring at MCS, including exchanges of ions and lipids, apoptosis and as the sites marking organelle fission (Wu et al., 2018). Recently, it has been shown that mitochondria-Rab7a endosome contact sites act as platforms for mRNA translation, which is crucial for mitochondrial function in retinal ganglion cells (RGCs) (Cioni et al., 2019). While mitochondria-ER contact sites (MERCs) dysfunction have not been studied directly in RGCs, fibroblasts from patients with Wolfram Syndrome caused by *WFS1* mutations, which is characterised by optic atrophy, diabetes and deafness, have been shown to have reduced MERCs compared with control fibroblasts (Angebault et al., 2018). Interestingly, manipulation of MERCs has also been shown to be neuro-regenerative *in vitro* and in animal models (Lee et al., 2019). Furthermore, deletion of the FFAT domain of protrudin, which is important for VAPA binding to ER MCS, has been shown to abrogate its regenerative effects (Petrova et al., 2020). To date, the quantification of MCS in axons has been largely limited to electron microscopy (EM techniques) and live-cell imaging of cultured neurons, with no alternative reliable method for analysing fixed optic nerve or peripheral nerve tissues directly.

The first description of MCS was in the 1950's based on EM. However, in the last two decades significant advances in imaging have enabled a much more detailed study of these membrane interactions. Advances in EM have led to 3D reconstructions of MCS using focused ion beam-scanning EM (FIB-SEM), permitting characterisation of ER-plasma membrane contacts, for example, in murine brain tissues (Wu et al., 2017). Another variant of EM, serial block face scanning EM (SBF-SEM) has previously been used on mouse optic nerves to examine MERCs in a model of hereditary spastic paraplegia, showing increased MCS in wild type compared to mutant (Yin et al., 2016). Similarly, SBF-SEM has been used to image MERCs in aged (12 months old) compared to young (1 month) murine optic nerves, finding comparatively reduced MCS in aged mice (Stahon et al., 2016). Whilst EM is considered as the “gold standard” for MCS characterisation, it is limited by high costs, time-consuming protocols and low throughput.

Confocal fluorescent microscopy has been widely used to visualise inter-organelle interactions in fixed and live cells. However, the spatiotemporal resolution of this technique is low and it does not allow the resolution of close membranes apposition. The subsequent development of super-resolution fluorescent microscopy (SRM) has significantly improved visualisation of MCS dynamics, including structured illumination microscopy (SIM), stochastic optical reconstruction microscopy (STORM), and stimulated emission depletion microscopy (STEDM). A number of important MCS have been elucidated with these techniques, including MERCs (Modi et al., 2019) and mitochondria-lysosome MCS driven by GTP bound lysosomal Rab7 (Wong et al., 2018). While SRM can be used in both live and fixed cell imaging, it is not possible to examine MCS in tissue samples such as the optic nerves.

Proximity-dependent fluorescent signal generation techniques utilise membrane markers, which if in sufficiently close apposition, reconstitute to emit a fluorescent signal. Proximity ligation assay (PLA) utilises complementary single-stranded DNA oligomers bound to secondary antibodies generated from different species that hybridise and complete a circular piece of DNA when the proteins of interest interact. The circular DNA can be amplified and detected by the addition of fluorescent probes (Fredriksson et al., 2002). MERCs have been widely studied with PLA in mammalian cell cultures to both detect and quantify these interactions (Angebault et al., 2018). Other fluorescence-based proximity-reporting techniques such as Fluorescence Resonance Energy Transfer (FRET), bimolecular complementation (BiC), dimerization-dependent fluorescent protein (ddFP), and a split GFP-based contact site sensor (SPLICS) (Cieri et al., 2018) have been developed, but these all require cellular or animal genetic modifications, which are not desirable when attempting to study protein-protein interactions in the context of gene therapy.

At present, there are limited methods available to quantify inter-organelle contact sites in optic nerve tissue either *in vivo* or *ex vivo* as RGC axons. Whilst it is possible to study primary RGCs, cultured RGCs only survive several days *in vitro*, making it very challenging to reliably assess MCS, particularly in regeneration experiments where the study of aged RGCs is necessary (Brown et al., 2015). Here, we present our experience of using PLA to quantify mitochondria-ER and mitochondria-Rab7 endosomes contact sites in uninjured optic nerve sections pre-treated with protrudin gene therapy, a therapy that has recently been shown to be neuroprotective and neuro-regenerative in the central nervous system (Petrova et al., 2020).

## 2 Materials and supplies

### 2.1 DNA constructs

Three adeno-associated virus (AAVs) vectors were generated for delivery to the retina by intravitreal injection: AAV2-GFP, AAV2-Protrudin-GFP (wild-type AAV2-Protrudin), and AAV2-phosphomimetic-Protrudin-GFP (active AAV2-Protrudin). The viral vector plasmid backbones (AAV2-sCAG-GFP) were kindly donated by Professor Joost Verhaagen (The Netherlands Institute for Neuroscience, Amsterdam, Netherlands). Protrudin-GFP was cloned from pEGFP-C1 plasmid into viral vector plasmids using Gibson cloning. Site-direct mutagenesis was performed in order to create the Protrudin phosphomimetic form (QuikChange II Site-Directed Mutagenesis Kit, Agilent Technologies). These constructs, including the DNA primers used for Protrudin cloning, have been described previously (Petrova et al., 2020).

### 2.2 Antibodies

Rabbit anti-IP3R-1 (Cell Signalling Technology, 8568, 1:300) binds the calcium channel inositol-1, 4, 5-triphosphate receptor 1 localized at the ER, mouse anti-VDAC1/Porin (Abcam, ab14734, 1:300) and mouse anti-TOM20 (BD Biosciences, 612278, 1:300) bind the voltage-dependent anion channel 1 and the mitochondrial importer submit TOM20 homolog, respectively, both located to the outer mitochondrial membrane, rabbit anti-Rab7 (Abcam, ab137029, 1:300) binds Rab7 GTPase located on late endosomes, and mouse anti-Grp75 (Abcam, ab2799, 1:300) binds the chaperone glucose-regulated protein-75 located at both the mitochondrial and ER when bound to VDAC1 and IP3R-1, respectively. Donkey anti-Mouse IgG (H + L) Alexa Fluor 568 (Thermo Fischer, A10037, 1:500), Goat anti-Rabbit IgG (H + L) Alexa Fluor 647 (Thermo Fischer, A-21244, 1:500).

The primary antibody pairs optimised for use with PLA presented herein were: 1) Anti-IP3R-1 and anti-VDAC1, and 2) Anti-TOM20 and anti-Rab7.

### 2.3 Proximity ligation assay

Duolink™ Proximity Ligation Assay (Sigma Aldrich, Misouri, USA) products were used: In Situ Red Starter Kit Mouse/Rabbit (DUO92101), In Situ Detection Reagents Far Red (DUO92013), Probe anti-mouse PLUS (DUO92001), and Probe anti-rabbit MINUS (DUO92005). The Starter Kit included: Blocking solution, antibody diluent, ligase stock (5X), amplification stock (5X), and polymerase (10U/μL), Buffer A (10 mM Tris, pH 7.4, 150 mM NaCl and 0.05% Tween) and Buffer B (200 mM Tris, pH 7.5, 100 mM NaCl). All reagents were stored at −20 °C and thawed over ice prior to use.

The kit is used by first staining cells or tissue of interest using two primary antibodies of interest raised in different species. A pair of semi-circular oligonucleotide-labelled secondary antibodies, each raised in the same species to the corresponding primary antibodies and labelled as PLUS or MINUS to distinguish the complimentary oligonucleotides that can hybridise (i.e. PLUS and PLUS or MINUS and MINUS oligomers cannot hybridise). If both primary antibodies are in sufficiently close proximity, hybridisation of the complimentary oligonucleotides occurs during ligation, whereby a circular DNA template forms and acts as a template for rolling-circle amplification. DNA polymerase then amplifies this signal up to 1000 times, whilst still bound to the antibody complex, permitting spatial localization. Detection oligomers coupled to fluorochromes hybridise to repeating sequences in the amplicon, allowing detection with fluorescent microscopy.

## 3 Detailed methods

### 3.1 Animal studies

All procedures were performed in accordance with protocols approved by the UK Home Office regulations for the care and use of laboratory animals under the UK Animals (Scientific Procedures) Act (1986), following ethical review by the University of Cambridge Animal Welfare and Ethical Review Body (AWERB). Animal work also met the requirements by the Association for Research in Vision and Ophthalmology's (ARVO) Statement for the Use of Animals in Ophthalmic and Visual Research. Female C57BL/6J mice aged 6–8 weeks (Charles River) were housed in a pathogen-free facility with free access to food and a standard 12-h light/dark cycle. All animals were housed at 18–23 °C and in 40–60% humidity. Intravitreal injections of viruses were administered 28 days prior to optic nerve dissection. Two microliters of the injecting solution for mice was drawn into a sterile 5 μL Hamilton syringe (#65RN; Needle: ga33, 8 mm, pst2, Hamilton Co.). Attention was paid to avoid lens penetration, extraocular muscles and vortex vein impingement. The Hamilton syringe was then held *in situ* for 30 s before a sterile 30-gauge needle (B. Braun Medical Ltd.) was used to puncture the central cornea, reducing intraocular pressure and injection solution reflux, at which point the Hamilton syringe was carefully withdrawn. Separate needles were allocated to each virus to prevent contamination, and syringes were rinsed between injections with ethanol followed by sterile phosphate buffer saline (PBS).

### 3.2 Optic nerve sections

Intraperitoneal sodium pentobarbital was used to sacrifice each animal, before transcardial perfusion with 4% paraformaldehyde (PFA). The optic nerves were carefully dissected from the skull base using a dissecting microscope and an external illumination source. Care was taken to preserve the optic chiasm, intra-orbital and intra-cranial segments of the optic nerves *in situ* before sharp and blunt dissection. The meningeal layers were removed carefully. The optic nerves were immediately placed in 4% PFA for 2 h before immersing in a freshly made 20% sucrose solution overnight. The optic nerves were then transferred into a freshly made 30% sucrose solution and stored at 4 °C. The optic nerves were embedded in Tissue-Tek OCT and snap frozen on a mounting stage using a Leica CM3050 cryostat. Sequential 14 μm longitudinal sections were collected on Superfrost microscopy slides and then dried and stored at −20 °C.

### 3.3 Immunostaining

• Humidity chamber
  1. A humidity chamber was prepared using a Western Blot Box (15.2 cm × 10.2 cm x 3.2 cm, XL size, Sigma Aldrich, Misouri, USA), lining the base with 2 layers of tissue (Kimberly-Clark™ Professional, 33670–04, Kimberly-Clark, Texas, USA) and a single layer of Parafilm® M (PM-999, Bemis Company, Wisconsin, USA). Instil Mili-Q® water was added to the base until the tissue was lightly soaked. For the following steps, unless stated otherwise, the incubation conditions are without agitation.
• Antibody staining
  2. Optic nerve sections were thawed on Superfrost slides in a staining trough at room temperature (RT) for 20 min (min) with agitation (Orbital Shaker, Standard 1000, setting 1, VWR International, Pennsylvania, USA).
  3. Optic nerve sections were then washed three times with phosphate buffer saline (PBS) for 15 min each with agitation using the same settings as above.
  4. The Superfrost slides were then dried carefully with Whatmann filter paper by tilting and tapping the edge of the slide, without making contact with the tissue.
  5. A hydrophobic pen (ImmEdge ® Hydrophobic Barrier PAP Pen, H-4000, Vector Laboratories, Burlingame, USA) was used to draw around the edge of each slide.
  6. Superfrost slides were then placed in the pre-prepared humidity chamber ensuring each slide was at least a slide width apart.
  7 40–50 μL of permeabilization solution 0.4% Triton X-100 in PBS was pipetted on each optic nerve for 120 min without compromising the fluid meniscus in contact with the hydrophobic marker.
  8. The blocking solution was aspirated and exchanged for Duolink™ PLA blocking solution for 60 min at RT.
  9. Duolink™ PLA blocking solution was aspirated. 50 μL of a mixture of two primary antibodies (1:300 dilution each), raised in different species, in Duolink™ antibody diluent was applied to each optic nerve section and stored overnight at 4 °C. In addition, a negative control was prepared for each experiment using a single primary antibody in a separate humidity chamber. This is to ensure that no cross-reactivity between the secondary antibodies occurred causing falsely positive PLA fluorescent signalling.
  10. The antibody mixture was aspirated and the slides washed with PBS three times for 15 min each with agitation using the same settings as above.
  11. 40 μL per optic nerve section of PLUS and MINUS antibodies diluted 1:5 with Duolink™ antibody diluent was prepared as follows: 8 μL PLUS antibody +8 μL MINUS antibody +24 μL Duolink™ antibody diluent.
  12. PLUS and MINUS antibody solution mixture was added to the optic nerve sections and incubated at 37 °C for 60 min.
• Ligation
  13. PLUS and MINUS antibodies solution was aspirated and the slides were washed with Buffer A twice for 5 min each.
  14. Ligation buffer was diluted in ddH_2_O 1:5 to make 40 μL per optic nerve section such that: 8 μL ligation buffer +32 μL ddH_2_O. This solution was added to the optic nerves and incubated at 37 °C for 30 min.
• Amplification
  15. Ligation buffer was aspirated and Buffer A was used to wash twice for 5 min each.
  16. Amplification buffer was diluted in ddH_2_O 1:5 to make 40 μL per optic nerve section such that: 8 μL ligation buffer +32 μL ddH_2_O
  17. Polymerase was added to the amplification buffer 1:80 and Incubated at 37 °C for 100 min.
• Final wash
  18. The amplification solution was aspirated and Buffer B was used to wash twice for 10 min each.
  19. Buffer B was aspirated and 0.01 X Buffer B added for 1 min.
• Preparation for imaging
  20. Buffer B was aspirated and Whatmann paper was used to dry the slides without making contact with the tissue samples.
  21. Slides were mounted with 4–5 drops of Fluorsave per slide, allow to dry at RT overnight and stored at 4 °C. All imaging was undertaken within 5 days to avoid fluorescence degradation.

### 3.4 Confocal microscopy

Images of immunostained fixed optic nerves were taken using the Zeiss LSM880 confocal microscope with Zen software (Zeiss, version 2.1 SP3) and equipped with the EC Plan-Neofluar 40x/1.30 Oil DIC M27 objective. Image dimensions were x: 1024, y: 1024, z: 12 pixels, 8 bit. Two laser channels were used with the same settings from the same experiment: 488 nm and 633 nm. The PLA signal was acquired by taking a z-stack of 12 images 0.80 μm thickness apart and an average intensity z-projection was created in Fiji software. These settings were adapted from previously established protocols in cell lines, where adjustments were made for the greater thickness of optic nerve sections and the need for larger z-stack intervals (Tubbs and Rieusset, 2016; Tubbs et al., 2014). PLA signal was quantified by taking an average threshold setting across each set of images and quantifying the total number of dots using the automated “Particle Analyzer” function on Fiji (Rasband, W.S. National Institutes of Health, Bathesda, MD, USA), setting the size (μm^2^) 0-infinity and circularity 0.00–1.00. Since PLA “dots” are not of a consistent size or shape, where a discrete single dot should be considered to represent two proteins’ close apposition or co-localization, we justify quantifying dots from 0 to infinity. The circularity setting is a generic measurement in ImageJ, in the “Particle Analyzer” tool, measuring how circular an object is, where 1.0 is a perfect circle and as the value approaches 0, it represents an elongated polygon. Where regions of interest (ROI) were selected, a 180.24 μm × 180.24 μm square (equivalent to 521 × 521 pixels) using the “Rectangle” function was used throughout the analysis.

### 3.5 Statistics and reproducibility

Statistical analysis was performed using GraphPad Prism version 8.4.0 (GraphPad Software, La Jolla, CA). The one-way ANOVA test was used with multiple comparisons to calculate statistical differences between treatment groups with Tukey's post hoc test. Graphs were produced using GraphPad Prism where statistical significance is indicated by: ns for non-significant, * for p < 0.05, ** for p < 0.01. *** for p < 0.001, **** for p < 0.0001.

### 3.6 Schematics

All schematics were created using BioRender software (Biorender, Toronto, Canada).

### 3.7 Results of experiment and discussion

It was recently demonstrated that increasing protrudin expression or activity mediates axon neuroprotection and neuro-regeneration *in vivo* in the adult optic nerve and retina, and *in vitro* in primary cortical neurons (Petrova et al., 2020). To further investigate the underlying mechanisms by which protrudin mediates these effects, we wished to examine whether upregulation of protrudin modulates MCS between mitochondria and other organelles. We focused our study on the interactions between mitochondria and the ER, and mitochondria and Rab7 late endosomes (LE) given that Rab7-mitochondrial MCS act as docking sites for mRNA translation in RGCs (Cioni et al., 2019), and three-way contact sites between ER-localized PDZD8/protrudin, Rab7 and mitochondria have been recently documented (Elbaz-Alon et al., 2020). We hypothesised that PLA, which is an established method for quantifying MCS, could be applied to examine fixed optic nerve tissues.

**Fig. 1:**
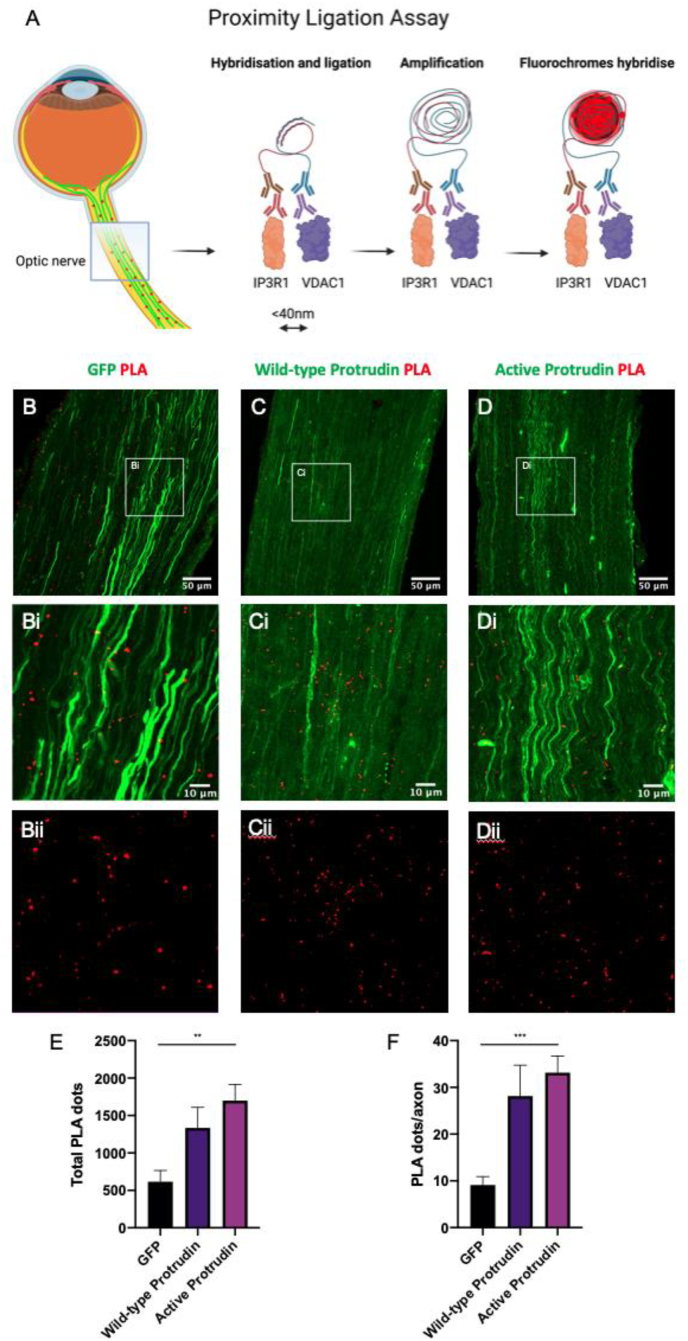
Schematic showing how fluorescent proximity ligation assay (PLA) dots (red) appear on optic nerve sections taken from mice treated with AAV2 gene therapy (transduced axons appear green) and a summary of PLA stages: primary antibodies rabbit anti-IP3R1 (red) and mouse anti-VDAC1 (purple) bind the target proteins, secondary antibodies with oligomers attached are introduced (anti-rabbit minus antibodies, brown, and anti-mouse plus antibodies, blue), if the IP3R1 and VDAC1 proteins are <40 nm in distance these oligomers hybridise and following ligation and amplification, fluorochromes hybridise to produce a discrete single dot on microscopy (A). IP3R1 and VDAC1 primary antibodies were used to co-stain experimental sections (B-D and Bi-Di), where GFP fluorescence, 488 nm, shows axonal transfection with AAV2 and far-red fluorescence, 633 nm, for PLA fluorescent signalling. Optic nerve sections (n = 3 mice for each group, from which 7 sections from each optic nerve were used for quantification, totalling 21 sections per group) harvested at day 28 following injection of control (B and Bi), wild-type protrudin (C and Ci), and active protrudin (D and Di). Far red channel alone, demonstrating PLA fluorescent dots (Bii-Dii). Quantification of total PLA dots (E) and PLA dots/axon (F) in each treatment group. (For interpretation of the references to colour in this figure legend, the reader is referred to the Web version of this article.)

Since PLA has become an established technique in cell biology and has been used successfully in retinal tissues (Caminos et al., 2015) and RGCs (Cioni et al., 2019) to examine protein-protein interactions, our aim was to apply it to optic nerve tissues to examine MCS. All the experiments were performed with a single antibody, VDAC1, used as control. PLA results of VDAC1 alone exhibited little to no PLA signal (Suppl. Fig. 1A-Aii), indicating the specificity of the method. In contrast, experiments using two antibodies, one labelling the ER and mitochondria, exhibited specific bright PLA foci (Fig. 1A). The mean total number (±standard error of the mean) of IP3R1-VDAC1 PLA fluorescent dots was 615 (±151) dots in optic nerve sections transduced with AAV-2GFP (control condition), 1334 (±274) dots in optic nerves expressing wild-type AAV2-protrudin and 1699 (±216) dots in optic nerves expressing active AAV2-protrudin (One-way ANOVA, p = 0.0033) (Fig. 1B–E). When controlling for the total number of PLA dots, and for the number of transduced axons in each section, the total number of PLA dots per axon was 9 dots/axon in optic nerve sections transduced with AAV2-GFP (control condition), 28 dots/axon in optic nerves expressing wild-type AAV2-protrudin and 33 dots/axon in optic nerves expressing active AAV2-protrudin (One-way ANOVA, p = 0.0008) (Fig. 1F). Negative controls demonstrated little to no PLA signal with anti-VDAC1 antibody alone (Suppl. Fig. 1A-Aii). Overall, the results indicate that MERCs increased significantly with wild-type and active protrudin gene therapy compared to the GFP control.

**Fig. 2:**
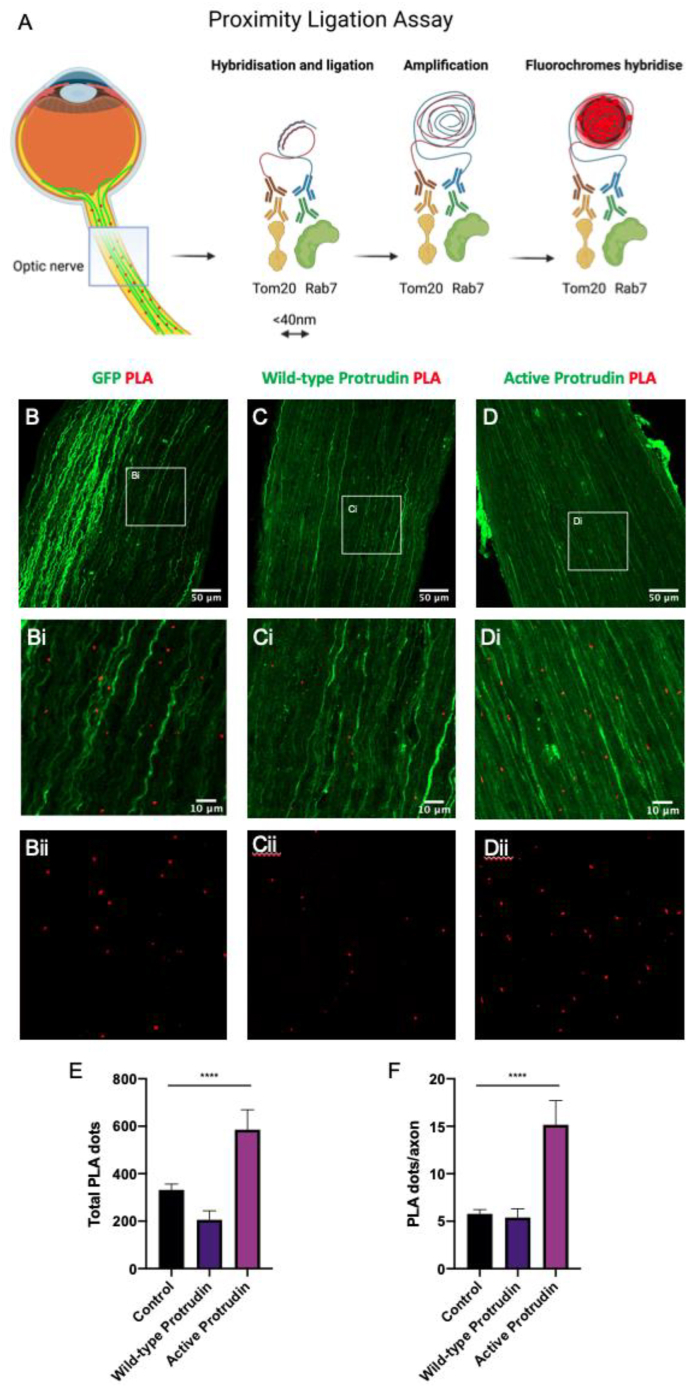
Schematic showing how fluorescent proximity ligation assay (PLA) dots (red) appear on optic nerve sections taken from mice treated with AAV2 gene therapy (transduced axons appear green) and a summary of PLA stages: primary antibodies mouse anti-TOM20 (yellow) and rabbit anti-Rab7 (green) bind the target proteins, secondary antibodies with oligomers attached are introduced (anti-mouse minus antibodies, brown, and anti-rabbit plus antibodies, blue), if the TOM20 and Rab7 proteins are <40 nm in distance these oligomers hybridise and following ligation and amplification, fluorochromes hybridise to produce a discrete single dot on microscopy (A). TOM20 and Rab7 primary antibodies were used to co-stain experimental sections (B-D and Bi-Di), where GFP fluorescence, 488 nm, shows axonal transfection with AAV2 and far-red fluorescence, 633 nm, for PLA fluorescent signalling. Optic nerve sections (n = 3 mice for each group, from which at least 7 sections from each optic nerve were used for quantification, totalling 24–28 sections per group) harvested at day 28 following injection of control (B and Bi), wild-type protrudin (C and Ci), and active protrudin (D and Di). Far red channel alone, demonstrating PLA fluorescent dots (Bii-Dii). Quantification of total PLA dots (E) and PLA dots/axon (F) in each treatment group. (For interpretation of the references to colour in this figure legend, the reader is referred to the Web version of this article.)

We then decided to examine MCS between mitochondria and late endosomes, given the emerging role of these interactions (Cioni et al., 2019; Wong et al., 2018). Using the same methodology, we used anti-TOM20 and anti-Rab7 antibodies to determine any change in MCS with WT and active protrudin constructs compared to GFP control (Fig. 2A). Further, we showed that wild-type and active protrudin increased mitochondrial-Rab7 LE MCS (Fig. 2E). All experiments were controlled with a single antibody, TOM20, which demonstrated little to no PLA signal (Suppl. Fig. 1B-Bii). The total mean (±standard error of the mean) TOM20-Rab7 PLA fluorescent dots was 331 (±31) in optic nerve sections transduced with AAV-GFP (control condition), 205 (±45) in optic nerves expressing wild-type AAV-protrudin and 585 (±58) in optic nerves expressing active AAV-protrudin (One-way ANOVA, p < 0.0001) (Fig. 2B–E). The total number PLA dots per axon was 5 dots/axon in optic nerve sections transduced with AAV-GFP (control condition), 5 dots/axon in optic nerves expressing wild-type AAV-protrudin, and 15 dots/axon in optic nerves expressing active AAV-protrudin (One-way ANOVA, p < 0.0001) (Fig. 2F). Negative controls demonstrated little to no PLA signal with anti-TOM20 antibody alone (Suppl. Fig. 1B-Bii). Overall, these results demonstrate that mitochondria-Rab7 LE MCS increased significantly with active protrudin gene therapy.

## 4 Pitfalls and trouble shooting

### 4.1 Background PLA staining

Significant background PLA staining can occur on the slide around the optic nerve. This can be avoided by paying particular attention to several steps of the protocol. First, sufficient washing must be carried-out throughout the protocol, where attention must be paid to aspirating as much of the previous solution as possible and replacing any damaged hydrophobic marker, which can break down and lead to leakage away from the optic nerve tissue. Second, the fluid meniscus at the interface of the hydrophobic marker must be maintained during washes to avoid cross contamination between samples. Third, fresh edges of Whatmann filter paper should only be used to dry areas around optic nerve tissue. Finally, multiple humidity chambers were used to accommodate separate control slides and different antibody combinations to prevent cross contamination.

### 4.2 Inconsistent PLA staining

This was found to occur when humidity chambers were not well sealed and undue drying of some samples had occurred. This can be ameliorated by using undamaged Western Blot Boxes and covering the top of the box with a single layer of tin foil. Wrapping the humidity chamber in multiple layers of tin foil can prevent the required rise in temperature, creating inconsistent PLA staining.

## 5 Conclusion

We have shown for the first time that PLA can be applied to fixed optic nerve tissues to examine MCS. Whilst we were not able to confirm our findings *in vitro* owing to the difficulty in culturing primary RGCs, patient iPSC-derived RGCs could provide a more reliable means to further elucidate MCS *in vitro*. PLA has previously been used in retinal cross sections to quantify interactions between KCNQ5-CaM, KCNQ5-VGluT1 and KCNQ5-GFAP (Caminos et al., 2015). However, these interactions were not specifically investigating MCS, rather, ion channel interactions with neurotransmitters and glial cells. To our knowledge, there has yet to be a reliable method to quantify MCS in ocular tissues from mammalian *in vivo* experiments without the need for EM. In this study, we demonstrate that PLA can be utilized for this purpose and we provide preliminary evidence that upregulation of protrudin expression may modulate mitochondria inter-organelle MCS.

## Contributions

J.C. conceptualised the contents of this paper in collaboration with J.P., P.Y.W.M., and R.E. JC and A.O. designed *in vivo* experiments. J.C. performed *in vivo* experiments. J.C. optimised the methods protocol for applying proximity ligation assay (PLA) to optic nerve tissue. J.C. performed validation, execution, curation, quantification and visualisation of PLA data. A.O. completed visualisation of schematics. J.P., P.Y.W.M., and R.E supervised the project and obtained funding. J.C. wrote the original draft of the paper. All authors provided comments on the manuscript and finalised the paper.

## Declaration of competing interest

The authors declare that they have no known competing financial interests or personal relationships that could have appeared to influence the work reported in this paper.
