# Supplementary material for: Quantifying inter-organelle membrane contact sites using proximity ligation assay in fixed optic nerve sections
Source: Exp Eye Res. 2021 Dec;213:108793. doi: 10.1016/j.exer.2021.108793 (PMC8683748; doi:10.1016/j.exer.2021.108793)
Supplement: Multimedia component 1 [file mmc1.docx]

**Supplementary figure**


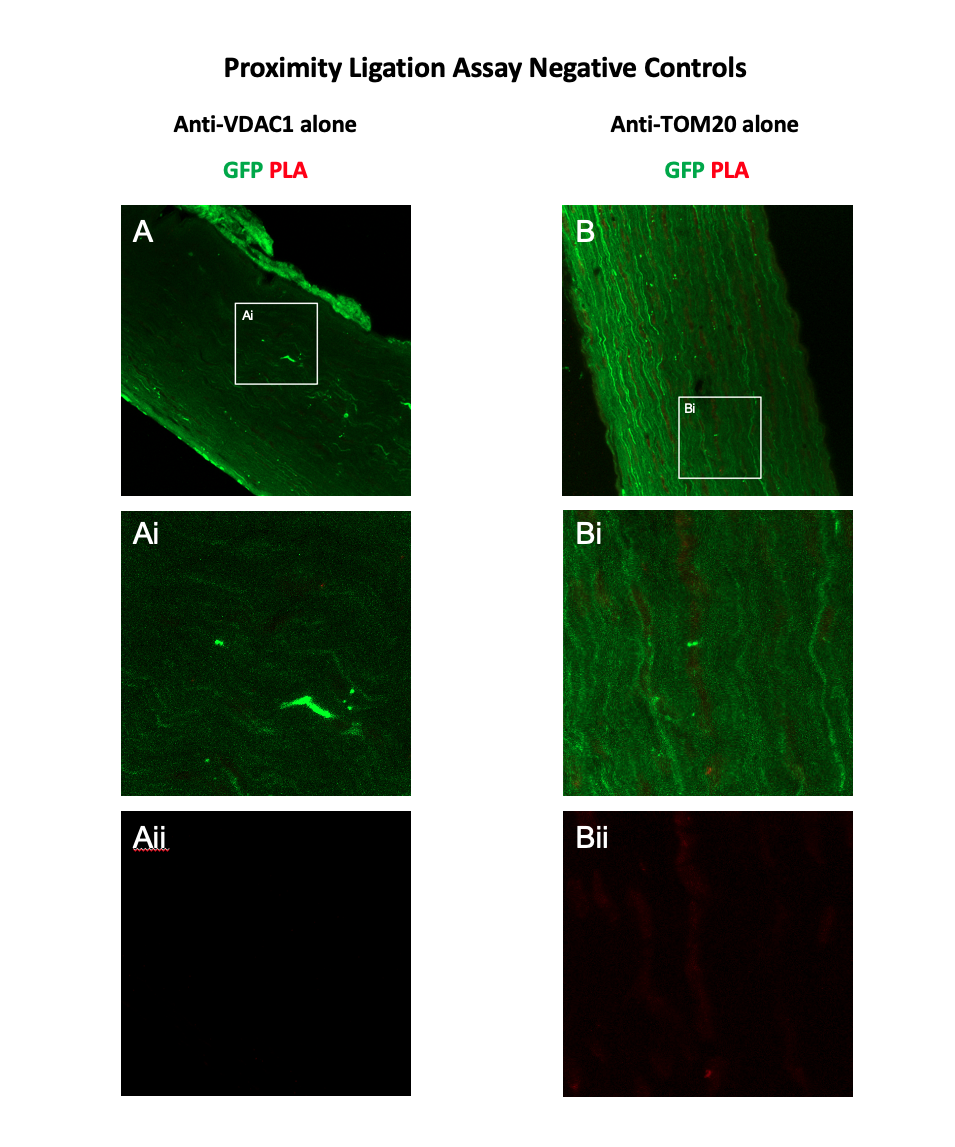


**Supplementary Figure 1**. Negative controls for PLA using a single antibody corresponding to the experiments represented in Figure 1 (A-Aii) and Figure 2 (B-Bii). GFP fluorescence, 488 nm, shows axonal transfection with AAV2 and far-red fluorescence, 633 nm, for PLA fluorescent signalling (A-Ai and B-Bi). The far-red channel alone demonstrates little to no PLA fluorescent dots when the anti-VDAC1 (Aii) or anti-TOM20 (Bii) antbody is used alone, demonstrating the specificity of the PLA assay.
